# A Review of the Effects of Abacus Training on Cognitive Functions and Neural Systems in Humans

**DOI:** 10.3389/fnins.2020.00913

**Published:** 2020-09-02

**Authors:** Chunjie Wang

**Affiliations:** Institute of Brain Science and Department of Psychology, School of Education, Hangzhou Normal University, Hangzhou, China

**Keywords:** abacus-based mental calculation, cognitive training, visuospatial processing, cognitive transfer, neuroplasticity

## Abstract

Abacus, which represents numbers via a visuospatial format, is a traditional device to facilitate arithmetic operations. Skilled abacus users, who have acquired the ability of abacus-based mental calculation (AMC), can perform fast and accurate calculations by manipulating an imaginary abacus in mind. Due to this extraordinary calculation ability in AMC users, there is an expanding literature investigating the effects of AMC training on cognition and brain systems. This review study aims to provide an updated overview of important findings in this fast-growing research field. Here, findings from previous behavioral and neuroimaging studies about AMC experts as well as children and adults receiving AMC training are reviewed and discussed. Taken together, our review of the existing literature suggests that AMC training has the potential to enhance various cognitive skills including mathematics, working memory and numerical magnitude processing. Besides, the training can result in functional and anatomical neural changes that are largely located within the frontal-parietal and occipital-temporal brain regions. Some of the neural changes can explain the training-induced cognitive enhancements. Still, caution is needed when extend the conclusions to a more general situation. Implications for future research are provided.

## Introduction

Research on the impacts of cognitive training on cognition and brain systems has long been of great interest to cognitive neuroscientists over the last decades. Many cognitive training programs have been shown to improve cognitive abilities (Diamond and Lee, 2011; Diamond, 2013). For instance, working memory training has been suggested to improve performance in untrained working memory (Holmes et al., 2009); video-game training has been reported to improve performance in visual attention and executive control (Strobach et al., 2012; Belchior et al., 2013); musical training and chess or go games playing that require a broad range of cognitive skills, have been found associated with superior performance in multiple cognitive tasks including working memory, executive control and reasoning (Kim et al., 2014; Benz et al., 2016; Burgoyne et al., 2016; Sala et al., 2017). Moreover, all the above cognitive training programs have been reported to produce functional and structural changes in the brain that may provide a neurophysiological basis for the cognitive transfer (Klingberg, 2010; Gong et al., 2015; Benz et al., 2016; Sohn et al., 2017). Although the findings are promising, it should be noticed that many existing training programs are constrained by the fact that they are conducted under controlled laboratory settings, which may pose difficulties in generalizing the obtained findings to broader contexts and real-world situations (Klingberg, 2010). In addition, some recent meta-analyses and reviews differ in their conclusions on the beneficial effects of many training programs including working memory training (Au et al., 2015; Melby-Lervåg et al., 2016), video-game training (Wang et al., 2016; Sala and Gobet, 2019), music learning (Benz et al., 2016; Sala and Gobet, 2017) and chess playing (Sala and Gobet, 2016, 2017). To date, the effectiveness of many existing training programs remains inconclusive. Other interventions for improving cognitive functions should be pursued.

**FIGURE 1:**
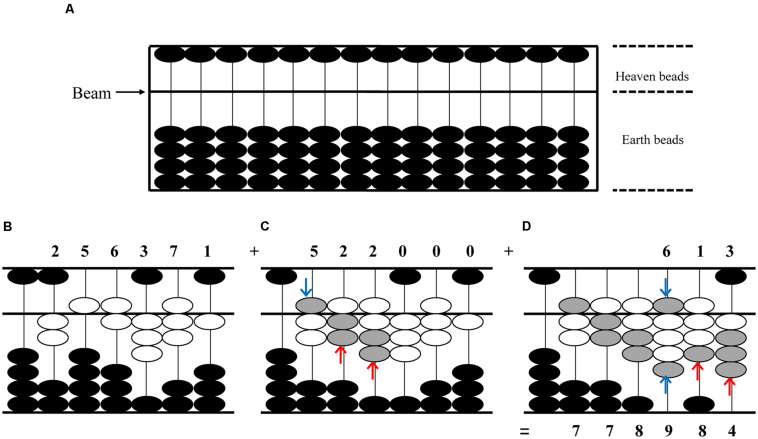
An addition example (256371 + 522613 = 778984) on the physical abacus. **(A)** A physical abacus that has not been operated. Dividing the upper and lower portion of the device is a horizontal bar called a beam. The beads below the beam are called earth beads, while the beads above the beam are called heaven beads. **(B)** The number 256371 is represented by the white beads on the abacus. One heaven bead represents a value of five when it is pushed down, and one earth bead represents a value of one when it is pushed up. **(C)** When adding 256371 to 522000 for the first step, the gray beads are pushed up and down with both hands simultaneously. The blue arrows mean operations with the left hand, while the red arrows mean operations with the right hand. **(D)** The second step of addition is adding the intermediated result to 613. The result 778984 is represented by both the white and gray beads.

Here, we focus on abacus-based mental calculation (AMC), a specific skill utilized for mental calculations. Abacus, a traditional calculation device that represents numbers by the visuospatial locations of beads (Figure 1), has been widely used in Asian countries to facilitate arithmetic operations including addition, subtraction, multiplication, division, and even root calculations. Importantly, skilled abacus players can perform fast and accurate arithmetic operations not only with the use of a physical abacus but also by the manipulation of an imaginary abacus in the mind (Frank and Barner, 2012). This skill is called AMC, which can be acquired through intensive training. During the training, students are first instructed to memorize the verbal principles of abacus operations; then they are trained to perform calculations by operating beads on a physical abacus; after long-term practice, they can get rid of the use of a physical abacus and learn to manipulate imaginary abacus beads in their minds by moving fingers in the air; finally, they are able to perform AMC as fast as possible without actual finger movements. Moreover, the abacus teachers can adjust the difficulty levels of the arithmetic practice in a step-by-step manner to help students gradually develop a high level of AMC capacity. For instance, AMC beginners are instructed to solve relatively easy arithmetic problems such as a list of 3 one-digit numbers, while experienced AMC users are trained to solve very complicated arithmetic problems such as a list of 10 four-digit numbers. Such an adaptive training on AMC can continuously challenge many cognitive processes, which may thus exert transfer effects to other cognitive abilities.

Recently, an increasing number of psychological studies have investigated the potential cognitive benefits of AMC training, suggesting that AMC training may be a promising tool in promoting cognition (Stigler, 1984; Miller and Stigler, 1991; Hanakawa et al., 2003; Chen et al., 2006; Lee et al., 2007; Ku et al., 2012; Tanaka et al., 2012; Yao et al., 2015; Wang et al., 2015, 2019; Weng et al., 2017; Zhou et al., 2019). Moreover, similar to other cognitive training programs, AMC training has been also found to produce functional and structural changes in the brain that may account for behavioral improvements in cognitive function (Hu et al., 2011; Wang et al., 2017; Weng et al., 2017; Zhou et al., 2019, 2020). Considering that the AMC training can be easily applied in school setting and daily life, it may show more advantages in promotion and application than many cognitive training programs constrained by a laboratory setting. Thus, findings in this field may be of great help for designing more practical cognitive interventions. However, before a more extended use that can be introduced into practice, we need to have a clearer and more systematic understanding of the cognitive and neural plasticity induced by AMC training. Additionally, although findings in this field are promising, a number of important issues remain uninvestigated. Therefore, this review study aims to provide an updated overview of the published works in this field to discuss the impacts of AMC training on cognitive functions and neural systems in humans, and to provide implications for future research.

## Cognitive Plasticity

### Mathematics

Mathematics has been considered as one of the most basic cognitive skills in numerate societies. Of particularly, mathematics at early period has been found to predict one’s future developmental outcomes including academic success (Hafer et al., 2002), career aspirations (Shapka et al., 2006) and economic incomes (Joensen and Nielsen, 2009). As pointed out in the introduction, AMC is an overlearned mental arithmetic skill based on manipulating beads on an imaginary abacus. Thus, it is reasonable to speculate that AMC training may lead to behavioral improvements in mathematics, especially in the arithmetic ability.

For instance, Hatano et al. (1977) reported that adult experts in AMC could mentally solve arithmetic problems involving numbers of more than 10 digits within seconds, which was far better than that of average control subjects. Similarly, child experts in AMC were reported to outperform their peers in various arithmetic computation problems (Stigler, 1984; Stigler et al., 1986; Amaiwa and Hatano, 1989; Chen et al., 2006). Several studies examined the processing mechanism of AMC and found that arithmetic performance of non-experts were highly disrupted by verbal distractors, while arithmetic performance of AMC experts was less affected by verbal distractors and much more affected by visuospatial distractors (Hatano et al., 1977; Frank and Barner, 2012). The authors concluded that the arithmetic advantages of AMC users were likely attributed to the implementation of a visuospatial imaginary strategy.

Notably, participants in these early studies were often self-selecting and highly motivated learners, who might be especially predisposed to perform arithmetic computations using a visuospatial strategy. This raises a question about whether AMC training is helpful for ordinary students in a standard classroom setting, or it is just suited to highly motivated individuals such as students with relatively high visuospatial skills. To address this issue, Barner et al. (2016) conducted a longitudinal training program in which 190 primary school students were randomly assigned into two groups who had received either 3 years AMC training or similar amounts of additional standard math curriculum. After 3 years of training, the study found significant improvements due to AMC training on a number of arithmetic measures, indicating that AMC training can be effectively implemented in standard classroom settings that can serve broad and representative groups of school-age children. Moreover, consistent with the hypothesis that AMC might be more beneficial to children with relatively high visuospatial skills, the study found that the effects of AMC training on arithmetic performance were significantly mediated by individual differences in visuospatial working memory at the beginning of AMC training.

In addition to the beneficial effects on arithmetic computation, two recent studies have claimed that AMC training may lead to behavioral improvements in visuospatial mathematical problems solving, given the visuospatial feature of AMC operations (Wang et al., 2015; Weng et al., 2017). For instance, in a previous study of Wang et al. (2015), 70 primary school students were divided into two groups who had received either 3 years AMC training or similar amounts of additional standard math curriculum. They found that AMC-trained children performed better than the control children not only on arithmetic computation problems, but also on visuospatial mathematical problems solving. The authors speculated that intensive training on manipulating an imaginary abacus might promote the construction and formation of visuospatial mental images, through which the training might generate a transfer effect to visuospatial mathematical problems solving. The findings have been replicated by a recent study with a larger sample size (Weng et al., 2017).

### Working Memory

Working memory is a multi-component system that supports the online storage and manipulation of task-relevant information. It provides a framework to support a wide range of cognitive activities such as language processing, problem solving and mathematics (Kane et al., 2007; Alloway and Alloway, 2010; Huettig and Janse, 2015). According to Baddeley’s theory, working memory includes three key components, namely visuospatial sketch, phonological loop, and central executive (Baddeley, 2012). Although AMC training is not originally designed as a working memory training program, many researchers argue that the operational processes of AMC involve multiple components of working memory and may thus exert a transfer effect (Lee et al., 2007; Dong et al., 2016; Wang et al., 2019; Zhou et al., 2020).

The visuospatial sketchpad refers to temporary storage of visual and spatial information. Functioning of this storage system is often assessed by simple span tests. As AMC operations involve temporary storage of imaginary beads in different visuospatial locations, AMC training may especially affect the visuospatial sketch. Several studies confirmed this hypothesis. For instance, Lee et al. (2007) and Chen et al. (2011) separately reported that children with 1 year AMC training performed better than their peers on different visuospatial memory span tests. Kamali et al. (2019) found that experienced AMC children (more than 3 years AMC training) showed an advantage over novices (less than half year AMC training) on three different visuospatial span tests. Bhaskaran et al. (2006) conducted a longitudinal study in which 100 primary school students received either 2 years AMC training or similar amounts of additional standard math learning. After training, the study found larger improvements on visuospatial span tests in the AMC group relative to the controls. All these studies indicate that AMC training may exert a transfer effect to the visuospatial sketchpad.

The phonological loop refers to temporary storage of phonological and auditory information. Regarding effects of AMC training on the phonological loop, several early studies have consistently reported that AMC experts exhibited higher digit spans than non-experts (Hatano and Osawa, 1983; Hatta et al., 1989; Tanaka et al., 2002). For instance, Hatano and Osawa (1983) showed that mean forward digit span of AMC experts was around 15, while for non-experts the digit span was about 7. Notably, the study also reported that digit span of AMC experts was more affected by concurrent visuospatial distractors while digit span of non-experts was more affected by concurrent verbal distractors. The authors concluded that AMC experts might have developed a visuospatial representation of numbers to expand their digit memory capacity. Subsequently, Hu et al. (2011) found that children with 3 years AMC training performed better than their peers in both digit and letter forward spans, indicating that the AMC training effects on phonological loop may be not limited to numerical information. Moreover, the advantage in letter forward spans has been replicated in two recent longitudinal studies examining the effects of 20-days AMC training on young adults (Dong et al., 2016; Zhou et al., 2020). During AMC training, students are usually instructed to memorize the verbal principles of abacus operations before they learn to perform calculations by manipulating a physical abacus. In addition, sequences of digits are often presented orally by abacus teachers. AMC learners need to continuously update the phonetic input of intermediate numbers, and then translate the phonetic representations of numbers into the visuospatial formats. Moreover, verbal retrieval of abacus principles is often involved to aid to visuospatial processing during AMC operations. These cognitive processes may gradually exert a transfer effect to the phonological loop. However, it remains unclear whether this beneficial effect could go beyond a placebo effect due to the lack of a comparable active control group.

The central executive control is a domain-general attentional control system interacting with the two storage systems. It includes three core components, namely memory updating, inhibition and mental-set shifting (Miyake et al., 2000). Given that AMC involves continuously updating of intermediate imaginary beads (Frank and Barner, 2012), AMC training may facilitate the memory updating ability. Consistent with this conjecture, Dong et al. (2016) found that 20 days AMC training improved visuospatial n-back performance in young adults. However, the study utilized a passive control group that could not rule out confounds from placebo effects. Importantly, Wang et al. (2019) also found positive effects of AMC training on visuospatial n-back performance when using an active control group, indicating that the transfer effect on memory updating can go beyond placebo effects. Moreover, previous work has suggested that AMC training enables users to simultaneously operate imaginary beads in different locations (Stigler, 1984). Thus, AMC training may enhance the shifting efficiency between different contexts and then exert a cognitive transfer to mental-set shifting. Two recent longitudinal studies confirmed this hypothesis by reporting that children with 3 or 5 years of AMC training performed better than active control groups in mental-set shifting tasks (Wang et al., 2015, 2017). Notably, both the n-back and mental-set shifting tasks in the above studies used visuospatial stimuli. It remains unclear whether similar effects are present for other stimuli such as phonological stimuli. Furthermore, there is another study reporting better response inhibition performance in AMC-trained children than their peers (Na et al., 2015). However, given that the central executive components are moderately correlated with one another (Miyake et al., 2000), the advantage in one central executive component may be driven by an advancement in another central executive component. Thus, a single task measure in one study is not enough to draw a clear conclusion. Future study should conduct a battery of cognitive tasks to address the effects of AMC training on each central executive component more rigorously.

### Numerical Magnitude Processing

The ability to efficiently process numerical magnitudes has long been considered as a cognitive foundation for the development of complex mathematics including arithmetic ability (Holloway and Ansari, 2009; Bartelet et al., 2014). Deficits in numerical magnitude processing are often observed in individuals with mathematical learning disabilities (Rousselle and Noël, 2007). Considering AMC users’ extraordinary gains in arithmetic ability, a question worth exploring is whether this arithmetic advantage is accompanied by an advancement in basic numerical magnitude processing.

A previous study by Yao et al. (2015) found that children with 2 years of AMC training performed better than their peers in a numerical magnitude comparison task, while there was no significant group difference in a physical size comparison task. This result suggests that AMC training has the potential to enhance efficiency in numerical magnitude processing but not in physical size processing. Besides, Wang et al. (2013) designed a numerical Stroop paradigm and found that compared to the controls, children with 3 years of AMC training were less affected by physical size information when they processed numerical magnitude information intentionally, while they were more affected by numerical magnitude information when they processed physical size information intentionally. These findings suggest that AMC-trained children are more able to access numerical magnitudes from numerical symbols both intentionally and automatically. According to several previous studies (Stigler et al., 1986; Miller and Stigler, 1991; Frank and Barner, 2012; Donlan and Wu, 2017), AMC users have been reported to be able to incorporate two types of symbolic number representations – visuospatial and phonological representations of numbers, and thus gain a more abstract and flexible understanding of numerical magnitudes. It is possible that a deeper understanding of numerical magnitudes endows AMC users with greater efficiency in accessing numerical magnitude from number systems both intentionally and automatically. Recently, Cui et al. (2020) found that AMC-trained children performed better than the controls in both number comparison and arithmetic tasks. The group differences remained significant after controlling for performance in a variety of cognitive tasks including visual perception, visuospatial processing, processing speed, working memory, language, attention, and general intelligence, indicating that the group differences could not be fully explained by differences in other cognitive abilities. Moreover, a mediation analysis showed that individual numerical magnitude processing partially mediated the group difference in arithmetic performance. These findings indicate that AMC training may directly enhance basic numerical magnitude processing efficiency, through which the training may contribute to arithmetic development.

Although the findings are very promising, the cross-sectional designs and passive control groups in the above three studies had resulted in limit meaningful conclusions. Further research with longitudinal designs and active control groups is needed to validate these findings.

### Fluid Intelligence

Fluid intelligence, which refers to the ability to reason and solve novel problems without the use of previously acquired skills and knowledge (Carpenter et al., 1990), is considered as one of the most important predictors for a wide variety of cognitive tasks (Gray and Thompson, 2004). Given the above positive findings of AMC on mathematics and working memory, and the fact that these two cognitive abilities are closely related to fluid intelligence (Unsworth and Engle, 2005; Primi et al., 2010), an intriguing question worth exploring is whether AMC training has the potential to improve fluid intelligence.

A previous study by Irwing et al. (2008) conducted an AMC training program in a large sample of 3185 children between 7 and 11 years. The AMC group received 34 weeks of AMC training while the control group received no training. After training, the Raven intelligence performance was significantly improved in the AMC group as compared to the control group. It has been suggested that Raven’s Progressive Matrices is largely a mathematical problem solving test in design format, which requires the application of several mathematical rules involving addition, subtraction and geometrical progression (Carpenter et al., 1990). Hence, the authors interpreted that AMC training gains in mathematical ability might indirectly improve performance on the Progressive Matrices test. However, this study did not include an active control group with alternative cognitive activities. While the use of a passive control group could eliminate test-retest effects, there was a possibility that the observed positive effect was accounted for by a placebo effect (Shipstead et al., 2012). Recently, Wang et al. (2019) conducted a 5 years longitudinal study in a sample of 144 primary school children. The participants were randomly divided into two groups who either received AMC training from the 1st to the 6th grade or received similar amounts of additional standard math learning. Although the calculation ability of AMC-trained children was far better than that of the control children, no significant group difference was found on the Raven’s intelligence scores. Thus, the effect of AMC training on fluid intelligence appears to not go beyond a placebo effect.

To date, although many studies have shown that cognitive training can improve performance in a variety of cognitive tasks (Diamond and Lee, 2011; Au et al., 2015; Benz et al., 2016), many other fail to replicate these positive effects, especially when it comes to improving domain-general cognitive abilities (far transfer) (Redick et al., 2013; Melby-Lervåg et al., 2016; Sala and Gobet, 2019). Of particularly, regarding the far transfer to fluid intelligence, existing evidence from many recent cognitive training studies (Redick et al., 2013; Thompson et al., 2013; Melby-Lervåg et al., 2016; Sala and Gobet, 2019), including AMC findings in the study of Wang et al. (2019), indicate very limited cognitive transfer. Thus, AMC training may be another example of cognitive training that shows benefits only in tasks tightly related to the trained tasks (near transfer). However, given that only a Raven test was utilized in prior AMC research to address potential far transfer (Irwing et al., 2008; Wang et al., 2019), further study is warranted. Like the work by Redick et al. (2013), future study should conduct a battery of cognitive tests comprising multiple measures of fluid or crystallized intelligence, reading comprehension, attention, verbal working memory, multitasking, perceptual speed, and more to address far transfer issues of AMC training more comprehensively.

## Neural Plasticity

The brain is not a static structure. An increasing number of neuroimaging studies have suggested the notion that cognitive training can result in substantial changes in functional activity and structure of the brain, which may contribute to cognitive benefits in the trained and untrained tasks (Klingberg, 2010; Gong et al., 2015; Benz et al., 2016; Sohn et al., 2017). AMC, a specific skill learning that involves the co-activation of multiple brain regions (Hanakawa et al., 2003; Chen et al., 2006; Ku et al., 2012), can also produce functional and structural changes in the brain that may account for AMC-related cognitive enhancements.

### Frontal-Parietal Regions

Frontal-parietal regions have been consistently reported to be activated by AMC operations (Hanakawa et al., 2003; Chen et al., 2006; Wu et al., 2009). Besides, AMC training has been found to induce functional changes in the frontal-parietal regions that may account for behavioral improvements in multiple cognitive abilities (Tanaka et al., 2012; Dong et al., 2016; Wang et al., 2017, 2019; Zhou et al., 2019, 2020).

Several research groups have utilized neuroimaging methods such as positron emission tomography and functional magnetic resonance imaging (fMRI) to examine brain regions engaged in AMC operations (Hanakawa et al., 2003; Chen et al., 2006; Wu et al., 2009; Ku et al., 2012; Du et al., 2013). All these studies indicate that AMC operations activate a bilateral frontal-parietal network that serves as the core substrate of visuospatial working memory, while conventional calculations rely on a language-related brain network including the Broca’s area. These findings are consistent with early psychological studies (Hatano and Osawa, 1983; Stigler, 1984; Hatano et al., 1987), suggesting that a visuospatial imaginary strategy has been developed by AMC training. Tanaka et al. (2002) further examined neural correlates underlying short-term digit memory in AMC experts. Similarly, they found that AMC experts activated the bilateral frontal-parietal brain regions that were considered as a visuospatial working memory network, while the non-experts recruited a language-related brain network including the Broca’s area. This study provides clear neurophysiological evidence that AMC experts can use a visuospatial representation of numbers for digit memory retention. It may be more efficient for AMC experts to maintain and manipulate numbers using a visuospatial representation than using a traditional phonological representation, and the bilateral frontal-parietal network may play a key role in these cognitive processes.

Notably, the conclusions of the above studies were made based on a direct comparison between AMC users and their peers. Although the activation differences could be explained by the employment of different behavioral strategies for the two groups, pre-training individual differences might have affected the results. Interestingly, a longitudinal fMRI study by Tanaka et al. (2012) addressed this issue more clear by examining recovery-related brain activity in an AMC user with a right hemispheric brain lesion. The participant had received 3 years of AMC training at an abacus school. After training, she kept using AMC in everyday activities, and became a finalist at a domestic abacus competition. In July 2009, the participant suffered from a right hemispheric infarct in the anterior and middle cerebral arteries. 6 months after her stroke, she reported that, although her knowledge of basic arithmetic facts and related operations of a physical abacus were intact, she could not use the visuospatial imaginary strategy for either mental arithmetic or digit memory. The first fMRI scanning was conducted at that time. Language-related brain activity including the Broca’s areas were observed during both mental arithmetic and digit memory tasks. Thirteen months after her stroke, she reported that she was able to shift the mental arithmetic strategy from linguistic to visuospatial representations, and her superior capacity for digit memory recovered. Then a second fMRI session was conducted. Interestingly, visuospatial-related brain areas including the bilateral frontal-parietal network were activated during both mental arithmetic and digit memory tasks. These findings extend previous cross-sectional studies and highlight the importance of the bilateral frontal-parietal network in AMC users’ superior arithmetic ability and digit memory.

It has been proposed that training of a certain neural circuit may lead to near transfer gains to other tasks that engage this circuit (Dahlin et al., 2008). Given the importance of the frontal-parietal network in AMC (Tanaka et al., 2002; Hanakawa et al., 2003; Chen et al., 2006), it is interesting to explore whether AMC training leads to functional plasticity in the frontal-parietal regions that may underlie near transfer to other cognitive abilities. In a longitudinal study by Dong et al. (2016), young adults were divided into two groups who had received either 20 days AMC training or no training. After training, activation of the frontal-parietal network during a n-back task decreased significantly in the AMC group but not in the controls. Moreover, the activation decreases were correlated with behavioral gains in the n-back task in the AMC group. In the literature, training-induced activation decreases have been suggested to be accounted for by enhanced neural efficiency in brain regions that are sensitive to cognitive training (Dux et al., 2009). It is thus plausible to speculate that frequent involvements of the frontal-parietal regions during training lead to enhanced neural efficiency of these areas, which may in turn facilitate memory updating. Similar results have been observed in another study (Wang et al., 2017). This study found that activation of the frontal-parietal network during a mental-set shifting task decreased significantly in the AMC-trained children, while activation in similar brain regions increased in the controls. Additionally, better mental-set shifting performance was correlated with lower frontal-parietal activation in the AMC-trained children but was associated with greater frontal-parietal activation in the controls. These findings provide further evidence that neural processes in the frontal-parietal regions may become more efficient as a function of AMC training. Prior work has reported that memory updating and mental-set shifting are moderately correlated with each another, indicating some common cognitive substrates between the two processes (Miyake et al., 2000). It is possible that the frontal-parietal regions serve as the common neural substrate of the two cognitive functions, and AMC training may improve both cognitive functions by enhancing neural efficiency of these regions.

Moreover, a recent study examined effects of AMC training on brain activation in a n-back task in children and reported an interesting Load-by-Group interaction (Wang et al., 2019). While lower activation of the frontal-parietal network in the AMC group relative to the controls was found at low memory loads, greater activation in similar regions in the AMC group than the controls was found at high memory loads. The authors speculated that the decreased activation at low memory loads might relate to more efficient neural processes during low cognitive-demanding tasks while the increased activation at high memory loads might be associated with an increasing ability to perform high cognitive-demanding tasks. Additionally, activation differences in the frontal regions were mostly seen at high memory loads while activation differences in the parietal regions were seen at both low and high memory loads. Thus, AMC-induced functional changes of the frontal region may be more pronounced when cognitive loads are high; in contrast, the parietal region may be the region where functional changes can be detected with tasks of low cognitive loads. Taken together, these findings suggest that functional changes following AMC training may be partly load-dependent.

Brain regions simultaneously activated in a given cognitive task are often functionally connected during resting-state (Sala-Llonch et al., 2012). Moreover, individual differences in task-induced brain activation can be partly predicted by individual differences in resting-state functional connectivity of same brain regions (Mennes et al., 2011). Given that prior research has consistently reported that AMC training induces activation changes in the frontal-parietal regions, Zhou et al. (2020) further examined whether AMC training would affect resting-state functional connectivity within the same brain regions. The study focused on three frontal and parietal regions that showed AMC-induced activation changes in a mental arithmetic task. Interestingly, after 20 days of AMC training, the average functional connectivity strength within the three brain regions were increased significantly in the AMC group while it remained stable in the controls, suggesting that AMC training may enhance the spontaneous communication within the frontal-parietal network. Further analysis showed that the increased functional connectivity in the AMC group was primarily driven by increased functional connectivity between the bilateral superior parietal lobules. This result is consistent with the findings by Wang et al. (2019), suggesting that the parietal regions may show functional changes that can be easily detected with tasks of low cognitive loads, even in the absence of task (e.g., resting-state). Additionally, a positive correlation between forward letter span and bilateral parietal functional connectivity was found in the AMC group at post-training session, but not in the controls. Given the functional role of superior parietal lobules in information maintenance (Koch et al., 2005), the beneficial effects on letter memory spans might be accounted for by enhanced neural communication between the bilateral parietal regions.

### Occipital-Temporal Regions

Apart from the frontal-parietal brain regions, the occipital-temporal regions have been also demonstrated to be involved in AMC operations (Hanakawa et al., 2003; Chen et al., 2006; Zhou et al., 2019). Moreover, long-term AMC training has been reported to produce structural and functional changes in the occipital-temporal regions that may explain training-induced cognitive benefits (Hu et al., 2011; Li et al., 2013; Weng et al., 2017; Zhou et al., 2019).

Hanakawa et al. (2003) reported that AMC operations in adults were associated with enhanced involvement of neural resources in the fusiform gyrus, which is an integral part of the ventral occipitotemporal junction. Moreover, activity of the fusiform gyrus was significantly correlated with the size of numerals involved in AMC operations. Given the functional role of the fusiform gyrus in the encoding and retrieval of figurative properties of visuospatial representations (Mellet et al., 1996), the authors speculated that this region might play a critical role in the visuospatial-dependent encoding and retrieval of imaginary abacus. Similarly, Chen et al. (2006) found that AMC operations in children were related to enhanced activation in the posterior temporal regions including the fusiform gyrus, providing further evidence that this brain region may be utilized to visually represent the imaginary abacus. The activation could not be simply attributable to basic perception of the visual number form, for similar activation was observed during an auditory AMC task.

Importantly, using voxel-based morphometry (VBM), Li et al. (2013) found that children with 3 years AMC training exhibited significantly smaller gray matter volume in the fusiform gyrus than the control children. This result provided further insight into the AMC-induced neural plasticity, for changes in brain morphology could be seen as one of the strongest evidences for the training-induced neural plasticity. The authors interpreted that gray volumes decrease in the fusiform gyrus were likely a consequence of neural pruning induced by frequent engagement of this region in encoding and retrieval of the imaginary abacus. Moreover, using diffusion tensor imaging (DTI), Hu et al. (2011) found that children with 3 years AMC training showed enhanced white matter fractional anisotropy in the occipitotemporal junction, a key pathway linking the fusiform gyrus and various cortical and subcortical regions. Additionally, individual differences in the white matter tracts were found positively correlated with children’s forward digit spans. Thus, long-term AMC training may enhance the integrity in white matter tracts related to encoding and retrieval of visuospatial information, which may contribute to digit memory retention using a visuospatial format.

Several recent fMRI studies have reported that the activation patterns in the occipital-temporal regions during untrained visuospatial working memory tasks were changed after AMC training (Dong et al., 2016; Wang et al., 2019; Zhou et al., 2019). For instance, a previous study by Dong et al. (2016) found that 20 days of AMC training on young adults not only led to activation decreases in frontal-parietal regions but also led to decreases in the occipitotemporal junction during a visuospatial working memory task. Moreover, activation decreases in the occipitotemporal junction were significantly correlated with performance gains of the n-back task in the AMC group. It was speculated that the frequent involvements of visuospatial encoding of numbers during AMC training might enhance neural processing efficiency of the occipitotemporal junction, which might enable the participants to perceive visuospatial information more efficiently.

Recently, accumulating evidence indicates that the human brain is topologically organized as a complex network (Rubinov and Sporns, 2010; Wang et al., 2020). Utilizing graph theory, Weng et al. (2017) examined effects of AMC training on resting-state network properties across the whole brain, and found that children with 1 year AMC training showed higher local efficiency or nodal degree in the right fusiform gyrus and bilateral superior occipital gyrus that are thought to be the primary neural substrates of visual processing. Additionally, local efficiency of the fusiform gyrus was found positively correlated with mathematical performance in AMC-trained children but not in the controls. Given the functional role of the visual cortical areas in visuospatial encoding (Engel et al., 1994; Malach et al., 1995), Li et al. (2013) argue that numbers may be transformed into a super-modal form of an imaginary abacus through the visual cortical areas especially the fusiform gyrus and then transmitted to high-order brain regions such as the frontal-parietal network for AMC operations. These processes may improve the capabilities of information transmission and processing of these visual-related brain areas in the whole functional brain network and in turn facilitate mathematical performance.

Taken the above studies together, there may be some coherence between functional and structural changes in the occipital-temporal regions that may ultimately drive AMC-related cognitive enhancements. Further study is warranted to verify this speculation.

## Conclusion and Future Directions

In summary, our review suggests that AMC training has the potential to improve cognitive abilities including mathematics, working memory and numerical magnitude processing. Considering that the operational processes of AMC require the integration of multiple cognitive processes including retrieval of abacus principles, math facts, number representation, and maintenance and manipulation of intermediate results (Stigler, 1984; Hanakawa et al., 2003; Frank and Barner, 2012), the positive effects of AMC on the above cognitive abilities may be attributed to a near transfer effect. Despite the few studies that investigated neural effects of AMC training, the findings indicate that AMC training produces functional and structural changes in the brain that are largely located in the frontal-parietal and occipital-temporal regions. Given that the frontal-parietal and occipital-temporal brain regions have been consistently reported to be activated by AMC tasks (Hanakawa et al., 2003; Chen et al., 2006; Ku et al., 2012; Zhou et al., 2019), AMC training may lead to near transfer effects to other cognitive tasks by impacting these brain regions. Additional evidence comes from several studies reporting that some of the AMC-related neural changes can be linked to training-related cognitive enhancements. These promising findings will be of great help for designing more effective cognitive interventions. In the following, we concluded with some considerations and directions for future research in this field.

First, experimental designs should be optimized in future AMC studies. Although previous AMC findings are promising, many AMC studies face methodological shortcomings that may cause too many possible confounds to allow the findings to be meaningfully interpreted. For example, many AMC studies utilized a single task measure to examine potential transfer effects. However, if one wishes to demonstrate the effectiveness of cognitive training on a cognitive ability, it is not enough to show enhancements on a single task measure. There are many factors unrelated to the targeted cognitive ability that may lead to behavioral improvements (Green et al., 2014). By contrast, if the training can improve performance on multiple task measures assessing the targeted cognitive ability and if the training effects remain significant after controlling for other cognitive performance, this will constitute much stronger evidence that the training indeed improves this cognitive ability. Besides, many AMC studies have utilized cross-sectional designs. Although the findings may be suggestive of a possible link between AMC training and cognitive ability, such designs cannot rule out confounds from pre-existing differences and do not allow causal inferences to be drawn. Moreover, other methodological limitations such as small sample sizes and the use of passive control groups may artificially inflate the overall effect of cognitive training (Sala and Gobet, 2019). Thus, future research should include a large battery of cognitive tasks, longitudinal designs with both pre- and post-training, large sample sizes, and at least one active control group to address the effects of AMC training more rigorously.

Second, potential factors that may moderate AMC training outcomes should be further scrutinized. Through the review about previous AMC studies, we have found that AMC-related cognitive or neural plasticity in children usually occurs after 1–5 years of AMC training (Lee et al., 2007; Chen et al., 2011; Weng et al., 2017; Wang et al., 2019). Notably, most past studies (Hu et al., 2011; Li et al., 2013; Wang et al., 2015; Barner et al., 2016; Kamali et al., 2019) have detected cognitive benefits or neuroplasticity after 3 years of AMC training – the amount of time when typical children can complete most existing AMC curricula. It seems that 3 years of AMC training may be the ideal period to produce cognitive benefits or neuroplasticity in children. However, more evidence is warranted to verify this speculation. Future research should compare training programs with different AMC training lengths to address this issue more rigorously. Moreover, two recent studies reported that training on AMC for as few as 20 days could improve working memory and alter the underlying neural correlates in young adults (Dong et al., 2016; Zhou et al., 2020). It seems that young adults spend shorter time in acquiring AMC-related cognitive and neural plasticity as compared to children. Given that the neural substrates supporting many cognitive functions are still developing until late childhood and adolescence (Giedd, 2004; Fair et al., 2007), it is possible that such cognitive or neural development during childhood may have buffered the effect of AMC training. Further research engaging similar AMC training in different age groups is needed to examine whether age would moderate AMC training outcomes. Furthermore, several other factors such as gender (Neubauer et al., 2010; Rojiani et al., 2017) and pre-existing individual differences (Bellander et al., 2011; Studer-Luethi et al., 2012) have been also identified as critical mediators for behavioral or neural effects of many cognitive interventions. Future studies should also consider whether and how these factors would mediate AMC training outcomes. Investigation of these factors would bring us a more complete understanding of the cognitive or neural plasticity induced by AMC training, and would help researchers design specific training programs that directly target particular cognitive abilities at an individual level.

Third, comparisons between AMC training and other cognitive training programs should be considered for future AMC research. Although several AMC studies have utilized active control groups in which participants were engaged in similar amounts of additional standard math curriculum and found beneficial effects of AMC training in boosting cognitive functions, we do not know whether AMC training shows advantages when compared to other cognitive training programs. For instance, both AMC and musical training are promising means to improve multiple cognitive abilities, and can be easily applied in school setting and daily life. However, no research has compared the effects of AMC and musical training on cognitive functions. Interestingly, we have noticed that AMC relies on the bilateral frontal–parietal regions that serves as the core substrate of visuospatial working memory (Hanakawa et al., 2003), while music recruits many brain regions related to phonological working memory and language processing (Koelsch et al., 2002). Hence, AMC training may have a greater impact on visuospatial working memory than musical training, while musical training may be more sensitive to phonological working memory than AMC training. Further research is needed to verify this speculation. Investigation of this question would bring us a better understanding of the effects of AMC training.

Fourth, the neural mechanisms by which AMC training may improve cognition remain largely unexplored, although the number of studies that have tried to identify these mechanisms has increased dramatically during the past decades. For instance, in contrast to extensive AMC studies examining the neural correlates of mathematics and working memory, relatively few AMC studies have examined neural underpins of numerical magnitude processing efficiency. Besides, most previous studies have focused on AMC-induced functional plasticity. Only two studies have investigated AMC-induced structural plasticity (Hu et al., 2011; Li et al., 2013) and no research has tested possible associations between functional plasticity and structural plasticity induced by AMC training. Moreover, most prior AMC studies have focused on isolated brain regions. Knowledge is still lacking regarding effects of AMC training on large-scale brain networks that may indicate massive changes in brain systems. Although two recent AMC studies examined resting-state functional connectivity (Weng et al., 2017; Zhou et al., 2020), they focused simply on task-free brain functional state and did not examine integration and interaction among large-scale brain networks. Investigation of large-scale brain network architecture during specific tasks may bring us a more complete understanding of the neural mechanisms underlying AMC-related cognitive transfer. Furthermore, existing AMC studies have mainly utilized unimodal approaches to investigate the neural plasticity. Although single imaging method can detect potentially important variations in the brain, each imaging modality has its own technical or physiological limits. Integration of different imaging modalities such as the integration of fMRI and simultaneous electroencephalography can help alleviate the limitations and yield a more complete understanding of the spatiotemporal dynamics of brain activity that ultimately drive cognition and behavior (Mulert et al., 2004). Therefore, combining multimodal data should be an important aspect of future AMC research.

Finally, effects of AMC training on special populations should be another important trend for future AMC research. Previous AMC studies have focused mainly on typical developing children and healthy adults. It remains unclear whether AMC training is helpful for special populations. For instance, children with mathematical learning disabilities often have difficulty in accessing numerical magnitude from numerical symbols (Rousselle and Noël, 2007; Iuculano et al., 2008). Interestingly, children with AMC training have been reported to be more able to access numerical magnitudes from numbers than their peers (Wang et al., 2013). Therefore, AMC training may be helpful in enhancing numeral-magnitude association in children with mathematical learning disabilities, and thus improve their overall mathematical performance. Moreover, during the past decades, physical abacus has been utilized as a computational aid for arithmetic operations in blind persons (Gissoni, 1963). AMC courses have been also introduced into the school math curriculum for blind children in many Asian countries (Becker and Kalina, 1975) and have been reported to be effective for overcoming many arithmetic computational problems encountered by the blind (Nolan and Morris, 1964). Thus, it is also interesting to explore whether AMC training is helpful in enhancing blind persons’ cognitive functions. Together, studies concerning the effects of AMC training on special populations such as children with mathematical learning disabilities and blind children are also important and should be fruitful.

In conclusion, the current review provides a brief summary for the existing literature about the effects of AMC training that we believe have yielded particular insights to the field of cognitive training. Still, a number of important issues remain uninvestigated, and we anticipate that future studies will solve these issues.

## Author Contributions

CW wrote the main manuscript text.

## Conflict of Interest

The author declares that the research was conducted in the absence of any commercial or financial relationships that could be construed as a potential conflict of interest.
