# Corrigendum: A Review of the Effects of Abacus Training on Cognitive Functions and Neural Systems in Humans

**DOI:** 10.3389/fnins.2021.788323

**Published:** 2021-10-19

**Authors:** Chunjie Wang

**Affiliations:** Institute of Brain Science and Department of Psychology, School of Education, Hangzhou Normal University, Hangzhou, China

**Keywords:** abacus-based mental calculation, cognitive training, visuospatial processing, cognitive transfer, neuroplasticity

In the original article, a funder was incorrectly omitted, the Starting Research Fund from Hangzhou Normal University (No. 2020QDL006) to Chunjie Wang.

The author apologizes for this error and states that this does not change the scientific conclusions of the article in any way. The original article has been updated.

## Publisher's Note

All claims expressed in this article are solely those of the authors and do not necessarily represent those of their affiliated organizations, or those of the publisher, the editors and the reviewers. Any product that may be evaluated in this article, or claim that may be made by its manufacturer, is not guaranteed or endorsed by the publisher.

